# ACKR2: An Atypical Chemokine Receptor Regulating Lymphatic Biology

**DOI:** 10.3389/fimmu.2016.00691

**Published:** 2017-01-11

**Authors:** Ornella Bonavita, Valeria Mollica Poeta, Elisa Setten, Matteo Massara, Raffaella Bonecchi

**Affiliations:** ^1^Humanitas Clinical and Research Center, Rozzano, Italy; ^2^Department of Medical Biotechnologies and Translational Medicine, Università degli Studi di Milano, Rozzano, Italy; ^3^Department of Biomedical Sciences, Humanitas University, Rozzano, Italy

**Keywords:** chemokine, chemokine receptor, atypical chemokine receptor, lymphatic vessels, inflammation

## Abstract

The lymphatic system plays an important role in the induction of the immune response by transporting antigens, inflammatory mediators, and leukocytes from peripheral tissues to draining lymph nodes. It is emerging that lymphatic endothelial cells (LECs) are playing an active role in this context *via* the expression of chemokines, inflammatory mediators promoting cell migration, and chemokine receptors. Particularly, LECs express atypical chemokine receptors (ACKRs), which are unable to promote conventional signaling and cell migration while they are involved in the regulation of chemokine availability. Here, we provide a summary of the data on the role of ACKR2 expressed by lymphatics, indicating an essential role for this ACKRs in the regulation of the inflammation and the immune response in different pathological conditions, including infection, allergy, and cancer.

## Introduction

### Chemokines and Chemokine Receptors

Chemokines are small chemotactic cytokines secreted by different cell types (e.g., immune cells, cancer cells, and endothelial cells) mainly involved in the regulation of immune cell migration during routine immune surveillance, inflammation, and development (1). According to the position of conserved cysteine residues in their N-terminus, chemokines are classified into four subfamilies: CXC, CX_3_C, CC, and C (2). In addition, they can be classified, on the basis of the conditions during which they are produced, in homeostatic or inflammatory chemokines (3, 4). Homeostatic chemokines (e.g., CCL19, CCL20, and CCL21) are constitutively produced and regulate leukocytes migration in basal conditions. Inflammatory chemokines (e.g., CXCL8, CCL2, and CCL3) are produced under pathological conditions, and they can act as secondary mediators induced by primary pro-inflammatory factors, such as IL-1 and TNF-α. Inflammatory chemokines actively participate in the inflammatory response attracting immune cells to the site of injury. Beyond their unequivocal role in regulating leukocyte recruitment, other activities, such as regulation of angiogenesis, fibrosis, proliferation, homeostasis, and cancer cell dissemination, have been attributed to chemokines (5–8).

The specific effects of chemokines on their target cells are mediated by chemokine receptors, members of a family of 7-transmembrane G-protein-coupled receptors (1). Chemokine receptors have a highly conserved structure, consisting of a single polypeptide chain with three intracellular and extracellular loops, an external N-terminus domain essential for the specificity of ligand binding, and an intracellular carboxy-terminus that, in concert with other motifs, such as the Asp–Arg–Tyr–Leu–Ala–Ile–Val (DRYLAIV) motif between the third transmembrane domain and the second intracellular loop, is involved in receptor signaling. As a general rule, chemokine binding to the receptor causes conformational changes that trigger intracellular signaling pathways involved in cell activation and migration toward increasing chemokine gradients (9). Depending on the type of chemokine they bind, chemokine receptors can be classified as CXCR, CCR, CX3CR, or XCR1.

Beyond canonical chemokine receptors, a smaller family of atypical chemokine receptors (ACKRs) has been identified (10). These receptors are called atypical because they share structural features with canonical chemokine receptors and bind ligands with high affinity, yet, they are not able to induce cell migration. Indeed, these receptors have an altered DRYLAIV motif and, differently from the canonical chemokine receptors, upon chemokine engagement, they do not induce any GPCR signaling. Rather, ACKRs internalize and transport chemokines to the degradative compartment, modulating chemokine concentration and bioavailability. The family of ACKRs includes four receptors named according to the new nomenclature: ACKR1 (previously called DARC), ACKR2 (D6), ACKR3 (CXCR7), and ACKR4 (CCX-CKR) (11).

### Role of Chemokine and Chemokine Receptor Expression in Lymphatic Function

Chemokine and chemokine receptors expressed by lymphatic vessels (LVs) have been mainly studied in the context of leukocyte traffic. Indeed, the lymphatic system represents an important transport network for leukocytes, in particular antigen-presenting cells that migrate, through afferent LVs, from the periphery to the lymph nodes (LNs). This trafficking is mainly dictated by CCL21. CCL21 is constitutively expressed by LEC and can be upregulated by inflammatory stimuli, such as TNF-α, which induce the release of CCL21 intracellular stores (12). CCL21 promotes the recruitment of CCR7-positive dendritic cells (DCs) but also of neutrophils and T cells to draining LNs (13–15).

Interestingly, lymphatics produce many other chemokines in a stimulus-specific manner, indicating that they can fine-tune leukocyte recruitment (16). CXCL12 and CX3CL1 were found to induce DC migration to LNs (17, 18) while the function of other inflammatory chemokines produced by inflamed LEC, such as CCL2 and CXCL8, is not fully understood (12, 19).

Besides, in keeping with chemokine receptor expression on cancer cells, leukocyte-like homing toward LVs and LNs plays an important role in promoting cancer cell migration and metastasis. For example, CCR7-positive cancer cells metastasize to LNs where CCL19 and CCL21, the ligands for CCR7, are produced (20).

In addition to producing chemokines, lymphatic endothelial cells (LECs) express canonical chemokine receptors. Primary culture of murine LEC was found to be positive for the expression of CCR5, CCR9, CXCR4, and CXCR6, whereas they weakly expressed CCR4, CCR6, CCR8, CCR10, CXCR3, and CX3CR1 (21). The function of these receptors expressed by LEC is still unknown, with the exception of CXCR4 that has a role in promoting lymphangiogenesis, similar to its angiogenic role in vascular endothelial cells (21).

Interestingly, LECs express ACKRs: ACKR1 is expressed by human podoplanin low LECs (22); ACKR2 by many human tissues on afferent lymphatic (23); ACKR3 by human LECs, with restricted expression in the tonsil and kidney and increased expression during renal allograft rejection (24); and finally, ACKR4 is expressed by LEC in the LN capsule (25, 26). Here, we are summarizing data on the expression and role of ACKR2 by LECs. Increasing evidence suggests a crucial role for this receptor in the regulation of inflammation and immune response (12, 27).

## ACKR2/D6

The atypical chemokine receptor ACKR2, also known as D6 or CCBP2, is a highly promiscuous receptor capable of binding the majority of inflammatory CC-chemokines (28). Initially, according to its “atypical” features, it was assumed that ACKR2 was a non-signaling chemokine receptor. However, it was later demonstrated that not only is ACKR2 capable of internalization and scavenging of its ligands but that it also activates a β-arrestin-dependent signaling pathway, promoting receptor internalization and recycling to the cell membrane (29, 30).

### ACKR2 Expression by Lymphatics

ACKR2 is expressed by trophoblasts in the placenta, by some leukocytes (31), and by LECs. Indeed, Nibbs et al., in a seminal paper (23), demonstrated that within non-inflamed tissues, human LECs, but not vascular endothelial cells, express ACKR2. Moreover, they found that the receptor was expressed on a subset of lymphatics, thus suggesting the existence of functional heterogeneity within the lymphatic vasculature. Specifically, ACKR2 expression was found in human skin sections only in afferent LVs, both in regions near the epidermis but also deeper within the dermis. ACKR2 was found in small lymphatics in the villi of small and large intestine and in the lamina propria mucosae of colon and large collective lymphatics located in the muscular layer. The appendix also showed ACKR2-positive lymphatics in lymphoid tissue of lamina propria and in the lamina muscolaris externa. ACKR2 was also detected in other secondary lymphoid organs such as tonsils, spleen, and LNs on sinus-like channels and vessels in the parafollicular areas of the tonsils and the red pulp of the spleen. ACKR2 was not detected in heart, kidney, liver, skeletal muscle, brain, cerebellum, pancreas, prostate, and thyroid, whereas it was found in liver, lung, and placenta, but not on LECs (23).

The regulation of ACKR2 expression in LECs was only studied *in vitro* using human dermal LECs. McKimmie et al. found that ACKR2 is upregulated by the lymphangiogenic cytokine vascular endothelial growth factor-D, by the immunosuppressive cytokine transforming growth factor-β, and by the inflammatory mediators IL-6, type-I IFNs, and IFN-γ. On the contrary, the pro-inflammatory cytokine IL-1α induced a significant downregulation of ACKR2 (32).

### The Function of ACKR2 Expressed by LECs

Extensive evidence indicates that ACKR2 is involved in the regulation of chemokine levels around afferent LVs and in the removal of chemokines from inflamed tissues, thus acting as scavenger and “gatekeeper” by limiting the access, the interaction, and the inappropriate accumulation of inflammatory leukocytes in the lymphatic system, in particular in the subcapsular sinus region of LNs (33, 34).

McKimmie et al. observed a variable expression of ACKR2 on individual cutaneous LVs and a biased distribution toward the luminal face of the LECs, thus suggesting an involvement of ACKR2 in suppressing inflammatory leukocyte binding to lymphatic endothelial surfaces (32). Specifically, they demonstrated that ACKR2 contributes to the efficiency of antigen presentation of DCs, which is crucial in maintaining immune surveillance. Indeed, ACKR2, by scavenging pro-inflammatory chemokines, suppresses inflammatory chemokine binding to the LEC surface, thus increasing the availability and contributing to selective presentation of CCR7 ligands that attract CCR7^+^ mature DCs (32).

**Figure 1:**
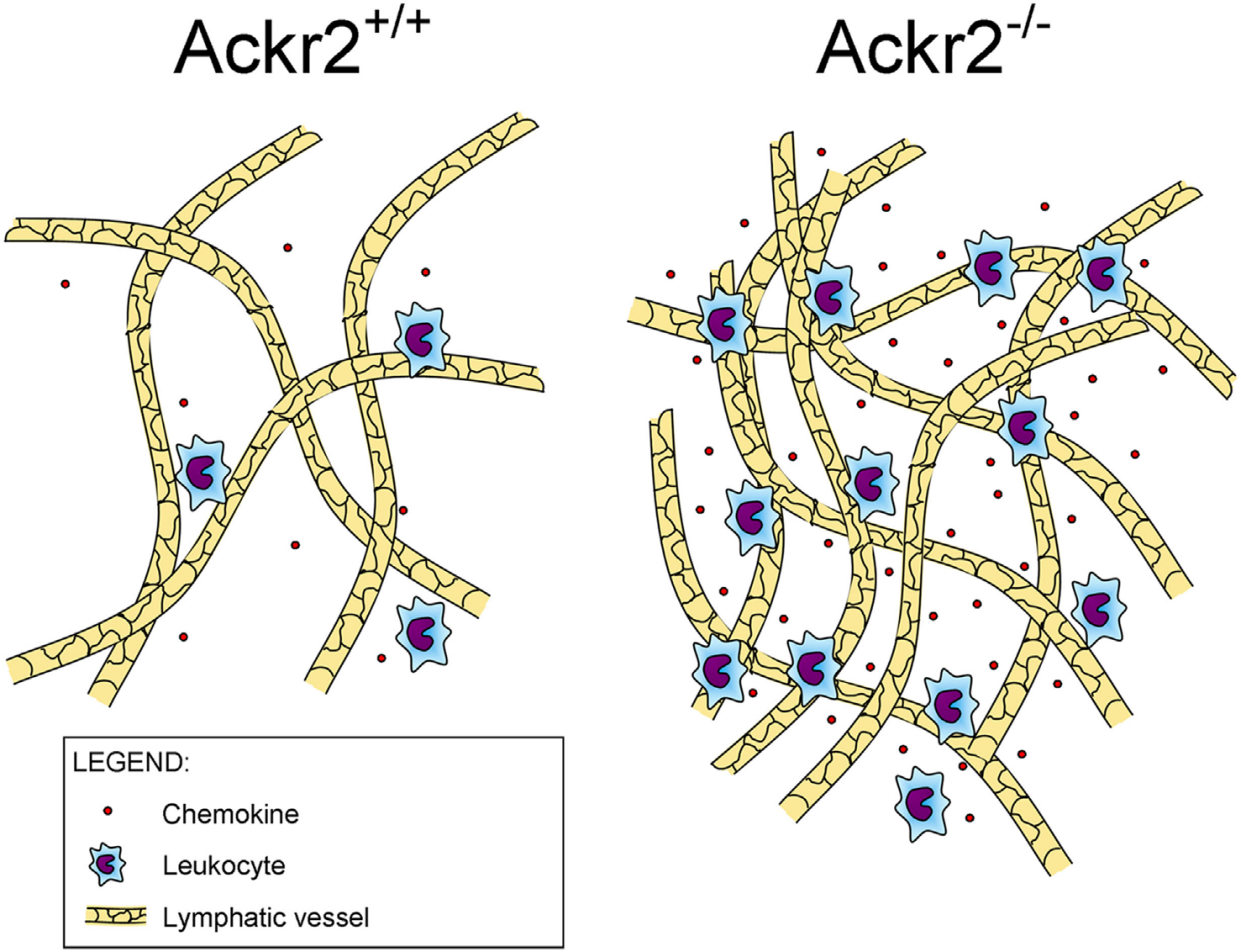
**Role of ACKR2 expression on lymphatic vessels**. ACKR2 expression on lymphatic endothelial cells, by scavenging inflammatory CC chemokines, shapes functional gradients promoting appropriate leukocyte recruitment into inflamed tissues, regulates their trafficking into lymph nodes, and controls lymphatic vessel density.

Recently, it was found that ACKR2 regulates LV density, competing with the canonical chemokine receptor CCR2 for the binding of CCL2, a chemokine produced in the skin during inflammation that drains into the LNs where it induces monocyte infiltration (Figure 1). By immunostaining of LV networks, Lee et al. found that *Ackr2^−/−^* mice displayed an increased LV density in the ears, diaphragms, and popliteal LNs in resting and regenerative conditions, compared to WT mice. Further investigation on E15.5 embryonic mice showed the presence of macrophages in proximity of developing LVs and involved in developmental lymphangiogenic processes. These macrophages, recruited by CCL2 and phenotyped as CD11b^high^ F4/80^low^ Lyve-1^+^, were significantly higher in *Ackr2^−/−^* mice whereas they were reduced in *Ccr2^−/−^* mice. Together, this evidence demonstrated that ACKR2 and CCR2 reciprocally regulate macrophage proximity to LVs and contribute to control lymphangiogenesis in inflammatory conditions. Accordingly, *Ackr2^−/−^* mice displayed relatively inefficient antigen presentation (35).

## Insights into Lymphatic ACKR2 Expression and Function During Disease

Several studies using ACKR2-deficient mice or human samples have demonstrated an important role of ACKR2 expressed by LVs in inflammatory conditions and in cancer. The general emerging picture is that ACKR2 acts as a negative regulator of inflammation, but contrasting results were published on its role in the control of adaptive immune responses and in autoimmune disease development.

### The Role of Lymphatic ACKR2 Expression in Inflammation

Using murine models of cutaneous inflammation, such as phorbol ester skin painting and subcutaneous injection of complete Freund’s adjuvant, it was demonstrated that *Ackr2^−/−^* mice develop an exacerbated inflammatory response with increased necrotic areas, angiogenesis, and a significantly higher leukocyte infiltration compared to WT mice (36–38). In both models, ACKR2 promoted the resolution of cutaneous inflammation by chemokine clearance.

The cutaneous lesions developing in ACKR2-deficient mice after phorbol ester skin painting resemble human psoriasiform pathology, indicating a possible role of ACKR2 in the control of this pathology (36). Using a psoriasis-like skin inflammation model induced by imiquimod, it has been shown that localized inflammation and IFN-γ induce the upregulation of ACKR2 in remote tissues that control the spread of psoriasiform inflammation inhibiting T cell epidermal influx (39). Similar results were found in psoriatic patient lesions in which ACKR2 is highly expressed in the surrounding skin in comparison with healthy controls, while it is downregulated in lesional and perilesional sites (36, 37, 40).

Several papers have also described an important role for ACKR2 expressed by lung LVs in the regulation of pulmonary inflammation. ACKR2 expression is upregulated in the LVs and in alveolar macrophages in the lung of patients with chronic obstructive pulmonary disease. In this context, the increased expression of ACKR2 could regulate the trafficking of leukocytes from the lungs to draining LNs (41, 42). ACKR2 was also found on LECs in lungs and LNs from patients with pulmonary tuberculosis (43). *Ackr2^−/−^* mice infected by *Mycobacterium tuberculosis* show reduced survival, compared to WT mice, due to an increased number of lung and LN-infiltrating mononuclear cells and an abnormal production of pro-inflammatory cytokines and CC chemokines (43). Interestingly, inhibition of inflammatory chemokines in *Ackr2^−/−^* mice led to less controlled growth of *M. tuberculosis*, indicating that ACKR2 has an important role also in immune activation. The role of ACKR2 in the lung was also studied in an allergen-induced airway disease model. Allergen-challenged ACKR2-deficient mice had more lung inflammation compared to WT counterparts, having more DCs, T cells, and eosinophils in the lung parenchyma and more eosinophils in airways. Surprisingly, ACKR2-deficient mice had reduced airway responses to methacholine compared to WT mice, indicating that ACKR2 has opposing effects on allergic inflammation and airway reactivity (44).

Contrasting results have been published on the role of ACKR2 expressed by LVs in intestinal inflammation. ACKR2 is overexpressed by LVs in the gut in inflammatory bowel disease, and it was found to have a protective role in a dextran sulfate sodium (DSS)-induced colitis mouse model (45). Indeed, *Ackr2^−/−^* mice have increased levels of inflammatory chemokines and infiltrating leukocytes, and increased intestinal inflammation, weight loss, and disease activity index, compared to WT mice (45). On the contrary, using the same murine model of intestinal inflammation, reduced clinical symptoms and tissue pathology in response to DSS in ACKR2 deficient were observed compared to WT mice. This protection is due to increased secretion of IL-17A by γδ-T cells in the lamina propria (46).

The role of ACKR2 in autoimmune diseases is also debated. ACKR2-deficient mice were described to be protected from the development of an experimental model of autoimmune encephalomyelitis (EAE) due to impaired migration of DCs and inhibition of T cell priming (47). More recently, using the same EAE model, it was described that ACKR2-deficient mice are not protected from the development of the disease but, on the contrary, they develop worse clinical symptoms compared to WT mice due to increased innate B cell-dependent production of IL-17 (48). Finally, in a murine model of graft versus host disease, we have found that ACKR2-deficient mice are protected from the development of the disease due to increased number of inflammatory monocytes with enhanced immunosuppressive activity (49).

In conclusion, ACKR2 expressed by LVs has an anti-inflammatory function by clearing chemokines present in inflamed tissues. This activity promotes the migration of DC to LN through LVs that is necessary for the induction of the adaptive response but that can be detrimental in autoimmune diseases. Conflicting phenotypes published could possibly be explained by the fact that ACKR2 is also controlling IL-17 production, a critical cytokine for inflammatory and autoimmune diseases.

### The Role of LV ACKR2 in Cancer and Metastasis

In human cancer lesions, ACKR2 was found to be expressed by peritumoral LVs in oral squamous cell carcinomas and in colon cancer. Accordingly, murine models of inflammation-induced cancer in the skin and in the gut revealed that ACKR2 protects mice from the development of tumors by dampening inflammation (38, 45).

ACKR2 was found to be protective in cancer progression also when expressed by tumor cells, by inhibiting inflammatory chemokines and protumoral leukocyte infiltration. ACKR2 is expressed by vascular tumors with lymphatic origin or differentiation (23) and is highly expressed by Kaposi’s sarcoma spindle cells (32, 50–52). In this latter tumor, we have found that ACKR2 expression is downregulated in more aggressive tumors by the activation of the KRAS/BRAF/ERK pathway, thus unleashing chemokine-mediated macrophage recruitment and their acquisition of an M2-like phenotype that sustains angiogenesis and tumor growth (52, 53).

ACKR2 was also found to be expressed in human breast cancer, and its expression predicts relapse-free survival (RFS) (54) while it is inversely correlated with axillary lymph node metastasis (55). Of note, a functional non-synonymous single nucleotide polymorphism of ACKR2 is associated with lymph node metastasis and RFS in breast cancer, indicating that the expression and function of ACKR2 in the host could also affect tumor progression (54, 56).

In conclusion, while it is clear that in inflammation-induced cancer ACKR2 expression by tumor cells inhibits cancer progression by decreasing macrophage infiltration and angiogenesis, further studies are necessary to understand the exact nature of the role of ACKR2 expressed by the host and how it can affect tumor progression and metastasis.

## Concluding Remarks

Lymphatic vessels have been traditionally considered as an inert drainage system, which passively transports fluids, proteins, and leukocytes. However, an increasing number of studies show that lymphatics play a much more active role, especially in the context of inflammation and ongoing immune responses. The expression of chemokine and chemokine receptors by LECs can be seen as evidence in support of an active role for lymphatics in regulating immunity. By the expression of ACKRs, LECs create and shape functional gradients of chemokines and modulate leukocyte recruitment. Moreover, they avoid inappropriate accumulation of chemokines and immune cells into inflamed tissues.

Here, we reported data demonstrating the essential role of ACKR2, expressed by LECs, in regulating chemokine concentration and leukocyte migration. This promotes the resolution of inflammatory responses in different pathological conditions including infection, allergy, and cancer. This evidence enables the speculation that ACKR2 could be considered as a potential therapeutic target to be induced in order to attenuate inflammation, e.g., during psoriasis and lung infection.

Even if the role of ACKR2 in inflammatory conditions has been clarified, further experimental studies are required to better understand its role in tumors. In this context, although an inverse correlation between ACKR2 expression and tumor stage was observed, it is unclear whether this correlation can be utilized as a clinical prognostic marker. Another challenging issue yet to be resolved is to understand whether ACKR2 could be a putative target for cancer immunotherapy. Indeed, it remains to be investigated if the activity of ACKR2 on lymphatics promotes or inhibits adaptive immune responses and whether ACKR2, by shaping chemokine gradients, can influence cancer cell dissemination to metastatic organs.

## Author Contributions

All the authors have contributed to this review by writing and critically evaluating the literature.

## Conflict of Interest Statement

The authors declare that the research was conducted in the absence of any commercial or financial relationships that could be construed as a potential conflict of interest.
