# Corrigendum: ACKR2: An Atypical Chemokine Receptor Regulating Lymphatic Biology

**DOI:** 10.3389/fimmu.2017.00520

**Published:** 2017-05-11

**Authors:** Ornella Bonavita, Valeria Mollica Poeta, Elisa Setten, Matteo Massara, Raffaella Bonecchi

**Affiliations:** ^1^Humanitas Clinical and Research Center, Rozzano, Italy; ^2^Department of Medical Biotechnologies and Translational Medicine, Università degli Studi di Milano, Rozzano, Italy; ^3^Department of Biomedical Sciences, Humanitas University, Rozzano, Italy

**Keywords:** chemokine, chemokine receptor, atypical chemokine receptor, lymphatic vessels, inflammation

**Missing Funding Number**

There is an error in the Funding statement. In the original article, we neglected to include the number of the funder Italian Association for Cancer Research, AIRC IG 2014—15438. The authors apologize for this error and state that this does not change the scientific conclusions of the article in any way.

## Conflict of Interest Statement

The authors declare that the research was conducted in the absence of any commercial or financial relationships that could be construed as a potential conflict of interest.

